# Molecular mechanisms of enhancing porcine granulosa cell proliferation and function by treatment *in vitro* with anti-inhibin alpha subunit antibody

**DOI:** 10.1186/s12958-015-0022-3

**Published:** 2015-04-09

**Authors:** Liuping Cai, Aidong Sun, Hui Li, Anastasia Tsinkgou, Jianning Yu, Shijia Ying, Zhe Chen, Zhendan Shi

**Affiliations:** Laboratory of Animal Breeding and Reproduction, Institute of Animal Science, Jiangsu Academy of Agricultural Sciences, Nanjing, 210014 China; Department of Life Science, Xijiao-Liverpool University, Suzhou, 215123 China

**Keywords:** Immunoneutralization of inhibin bioactivity, Porcine granulosa cells, Estradiol secretion, Proliferation, Molecular mechanisms

## Abstract

**Background:**

This study was conducted to clarify the effect of the inhibiting action of inhibin on porcine granulosa cell proliferation and function, and to investigate the underlying intracellular regulatory molecular mechanisms.

**Methods:**

Porcine granulosa cells were cultured *in vitro*, and were treated with an anti-inhibin alpha subunit antibody, with or without co-treatment of follicle-stimulating hormone (FSH) in the culture medium.

**Results:**

Treatment with anti-inhibin alpha subunit antibody led to a significant increase in estradiol (E2) secretion and cell proliferation. Anti-inhibin alpha subunit antibody worked synergistically with FSH at low concentrations (25 microg/mL) to stimulate E2 secretion, but attenuated FSH action at high concentrations (50 microg/mL). Immunoneutralization of inhibin bioactivity increased FOXL2, Smad3, and PKA phosphorylation, and mRNA expression of the transcription factors *CEBP* and *c-FOS*. The expression of genes encoding gonadotropin receptors, *FSHR* and *LHR*, and of those involved in steroidogenesis, as well as *IGFs* and *IGFBPs*, the cell cycle progression factors cyclinD1 and cyclinD2, and the anti-apoptosis and anti-atresia factors *Bcl2*, *TIMP*, and *ADAMTS* were upregulated following anti-inhibin alpha-subunit treatment. Treatment with anti-inhibin alpha subunit down regulated expression of the pro-apoptotic gene encoding caspase3. Although expression of the pro-angiogenesis genes *FN1*, *FGF2*, and *VEGF* was upregulated, expression of the angiogenesis-inhibiting factor *THBS1* was downregulated following anti-inhibin alpha subunit treatment.

**Conclusions:**

These results suggest that immunoneutralization of inhibin bioactivity, through augmentation of the activin and gonadotropin receptor signaling pathways and regulation of gene expression, permits the development of healthy and viable granulosa cells. These molecular mechanisms help to explain the enhanced ovarian follicular development observed following inhibin immunization in animal models.

## Background

The hormone inhibin is a dimeric glycoprotein that is primarily secreted by the gonads and plays important roles in the regulation of reproductive activities in animals [1]. Inhibin inhibits the pituitary secretion of follicle-stimulating hormone (FSH) through negative feedback regulation [2,3]. Furthermore, inhibin derived from dominant or large mature follicles is believed to inhibit the development of subordinate follicles through a paracrine effect, representing a mechanism of follicular selection. Through these central and local actions, inhibin is involved in follicular selection and determination of the ovulation rate [4]. In this regard, inhibin has been viewed as a negative regulator of ovarian follicle development [5]. This response has been exploited in inhibin-immunized animals in an attempt to neutralize the biological activities of inhibin and to stimulate ovarian follicular development. Such treatments have resulted in enhanced ovarian follicular development [6], ovulation rate [7], litter size, and embryo quantity and quality following superovulation [8-10]. The stimulation of follicular development by inhibin immunization is accompanied by enhanced secretion of estradiol (E2) [7,9,10], although its role in the elevation of pituitary FSH secretion remains unclear [11]. This suggests that immunoneutralization of inhibin bioactivity may directly stimulate follicular or granulosa cell function, apart from the stimulation induced by enhanced FSH secretion. This notion is supported by evidence of direct stimulation of E2 secretion by cultured bovine granulosa cells treated with an antibody against the inhibin α-subunit [12]. Apart from enhanced E2 secretion, the mechanism underlying the enhanced growth and development of ovarian follicles following inhibin immunization remains unknown. Therefore, this study was performed to evaluate the molecular regulation of granulosa cell function by investigating the proliferation of E2-secreting cells and the expression of key molecules involved following the treatment of cultured porcine granulosa cells with anti-inhibin α-subunit antibody.

## Methods

### Granulosa cell culture

Ovaries of prepubertal gilts aged 165–180 days were obtained from a local slaughterhouse and transported to the laboratory in a vacuum thermos flask in sterile physiological saline at 30–37°C within 2 h of isolation. After ovaries were washed three times with sterile physiological saline at 37°C, follicular fluid and granulosa cells were aspirated from 40 medium-sized follicles, between 4 and 6 mm in diameter, that contained clear follicle fluid, by using a 10-mL syringe. The cells were then transferred to a 15-mL centrifuge tube, and 1 mL of 0.25% trypsin was added to digest cell lumps. Following incubation at 37°C for 3–5 min to disperse clumps of cells, 1 mL of 10% fetal calf serum-supplemented Dulbecco’s modified Eagle’s medium/Ham’s F-12 nutrient mixture (DMEM/F12, without phenol red) was added to the tube to terminate trypsin digestion. The cells were then centrifuged at 800 *g* for 15 min to be precipitated and then washed twice with phosphate-buffered saline (PBS).

Cell density was adjusted to 1 × 10(5) cells per well in a 96-well plate, in 200 μL of culture medium containing 10% fetal calf serum (FCS), and cell survival rate was measured by the trypan blue exclusion test, which was 66 +/− 7%. The cells were incubated under a humidified atmosphere containing 5% CO_2_ at 37°C for 24 h, and then washed with PBS to remove any unattached cells. Then, the culture medium was changed and replaced with new DMEM/F12 medium containing 2% FCS, 0.1 μM androstenedione, and a polyclonal anti-inhibin α-subunit antibody at a final concentration of 0, 25, 50, 100, 200, or 300 μg/mL. For the FSH treatment, different concentrations of FSH (0, 10, 20, or 50 ng/mL) were added to the medium, or combined with different doses of anti-inhibin α-subunit antibody (0, 25, 50, or 100 μg/mL). The plates were incubated for a further 48 h under a humidified atmosphere at 5% CO_2_ and 37°C. At the end of each incubation period, aliquots of the culture medium were collected for measuring E2 concentration. Each treatment combination was replicated in 3 culture wells in one experiment, and each experiment was repeated by 6 times.

For measurements of mRNA gene expression and phosphorylated protein levels, cells were plated at a density of 2 × 10(6) cells per well in a 6-well plate, in 2000 μL of culture medium (DMEM/F12 containing 10% FCS). Following the initial culture for 24 h and washing with PBS as described above, cells were further cultured for 48 h in the DMEM/F12 medium containing 2% FCS, 0.1 μM androstenedione, and an anti-inhibin α-subunit antibody [6], at a final concentration of 0, 50, and 200 μg/mL, respectively. Then, the culture medium was carefully aspirated out, and 200 μL TRIzol (Invitrogen), for RNA extraction, or radio-immunoprecipitation assay buffer (RIPA; Beyotime Institute of Biotechnology) supplemented with 0.1% sodium dodecyl sulfate (SDS), for protein extraction, was added to each well. After digestion of the cells for 5 min, lysates were aspirated and stored frozen at −80°C until further analysis.

### Measurement of E2 concentration

Concentrations of E2 in the culture medium were measured by enzyme-linked immunosorbent assay (Beijing North Institute of Biological Technology; Beijing, China), according to the manufacturer’s instructions. The standard curve ranged from 40 to 1000 pg/mL for E2. Conditioned supernatants were diluted in FCS-free medium. Depending on the E2 concentration in the conditioned supernatants, samples were diluted 5, 40, 120, and 200 times, respectively, for treatment with each antibody concentration to ensure that the final value fell within the detection range of the standard curve. Each sample was assayed in duplicate, and the E2 concentration was calculated by multiplying the end value by the dilution factor. The assay sensitivity, range, and intra-assay coefficient of variation were 1 pg/mL, 1–1000 pg/mL, and <15%, respectively. All samples were used in a single assay.

### Measurement of cell proliferation

3-(4,5-dimethylthiazol-2-yl)-2,5-diphenyl-tetrazolium bromide (MTT) is converted into yellow formazan after being reduced by succinate dehydrogenase, which is synthesized by the mitochondria of live cells. Therefore, the production of formazan is proportional to the number of live cells, which was used to represent granulosa cell proliferation in the present study. Following granulosa cell culture as described above for 24, 48, and 72 h, 10 μL of MTT solution containing 5 mg/mL thiazolyl blue MTT was added to each well of Cell Counting Kit 8 (Shanghai QCBio Science & Technologies CO., Ltd.; Shanghai, China). The cells were cultured for a further 3 h and the optical density (OD) of the yellow color was measured at 490 nm by using a Biotek EON microtiter plate reader. Each treatment was repeated by 2 duplicate wells, and then repeated by 6 independent experiments.

### Western blotting

RIPA lysates from 2 culture wells of the same antibody treatment were combined and centrifuged for 15 min. The pellet was discarded and the protein concentration in the supernatant was measured by the BCA assay (Applygen Technologies, Inc.; Beijing, China). Thirty micrograms of protein-lysate from each sample was loaded and separated by 12% SDS-polyacrylamide gel electrophoresis, and proteins were transferred onto polyvinylidene fluoride membranes. The membranes were then blocked using 5% (w/v) fat-free dry milk/Tris-buffered saline (TBS) at 4°C overnight, and then washed with TBS/0.05% Tween 20 three times for 20 min each. Membranes were then incubated with primary antibody against phosphorylated forms of Smad3 (Santa Cruz Biotechnology, Inc.), PKA (Millipore), and FOXL2 (Bioss), or β-actin (13E5) (Cell Signaling Technology, Inc.; Boston, MA) for 2 h at room temperature, and washed three times for 20 min each. The membranes were incubated with a goat anti-rabbit IgG-horseradish peroxidase-conjugated secondary antibody (Santa Cruz Biotechnology, Inc.) diluted 1:5000 in 5% (w/v) fat-free dry milk/TBS for 1 h at room temperature. Membranes were washed three times and signals were visualized using SuperSignal West-Pico kit (Thermo Fisher Scientific; Waltham, MA, USA). The band intensity was normalized to that of β-actin. The final result was calculated as the mean of 4 independent cell cultures.

### Measurements of gene expression levels

Real-time quantitative polymerase chain reaction (qPCR) was performed to quantify the mRNA expression levels of β-actin*,* cytochrome P450 aromatase *(P450arom),* cholesterol side-chain cleavage cytochrome P450 *(P450scc),* steroidogenic acute regulated protein *(StAR),* inhibin-α, inhibin-β, *FSHR, LHR,* fibroblast growth factor *(FGF2),* vascular endothelial cell growth factor *(VEGF),* thrombospondin 1 *(THBS1),* insulin-like growth factors (*IGFs), IGFBPs,* CyclinD1, CyclinD2*,* cyclin-dependent kinase inhibitor 1B *(P27Kip), BCL,* caspase3, A disintegrin and metalloproteinase with thrombospondin motifs *(ADAMTS),* tissue inhibitor of metalloproteases *(TIMP),* forkhead box L2 *(FOXL2),* CCAAT/enhancer binding proteins *(CEBP),* and FBJ murine osteosarcoma viral oncogene homolog *(FOS)* in the cultured granulosa cells (Table 1). Total RNA was extracted from the TRIzol lysate, combined from 3 culture wells for each antibody treatment, and was reverse-transcribed using ReverTra Ace qPCR-RT Kit (Toyobo; Osaka, Japan) to generate template cDNA. PCRs were carried out in a 50-μL reaction volume containing SYBR Green I Master Mix (Toyobo) and 2.5 pmol relevant primers set as indicated in Table 1. An ABI PRISM_7500 sequence detection system (Applied Biosystems; Foster City, CA, USA) was used to detect the amplification products. Upon completion of the real-time qPCR, threshold cycle (Ct, defined as the cycle at which a statistically significant increase in the magnitude of the signal generated by PCR was first detected) values were calculated by sequence detection software SDS Version 1.2.2 (Applied Biosystems; Foster City, CA, USA). The levels of gene expression were expressed in the form of 2-△△Ct and normalized to expression levels of the β-actin internal housekeeping gene. Gene expression for each antibody treatment was calculated as the mean of 2 duplicate wells in a single culture, which each experiment was repeated by 6 times.

**Table 1:** **Primers used in the real-time quantitative PCR assay of genes**

**Gene**	**Accession number**	**Primer sequences (5′-3′)**	**Length (bp)**
β*-actin*	L08165	upstream: CCGAGAGAGAAATTGTGCGTGAC	166
		downstream: TCGGGGCACCTGAACCTCTC	
*P450arom*	NM_214429.1	upstream: GGTCACAACAAGACAGGA	168
		downstream: AACCAAGAGAAGAAAGCC	
*P450scc*	L34259.1	upstream: CCTGGGGGAGATAACGGTG	112
		downstream: ATGCGGAAGGCGGGGCTG	
*StAR*	AY368628.1	upstream: CATTACCATCTACTCCCAGC	109
		downstream: AACCCGTATCTTTCTTGTCAG	
*FSHR*	NM_214386.2	upstream: GCCCAGAACTAAAACACAATG	107
		downstream: TATAGACAAGTAACCGTCAGC	
*LHR*	JN120797.1	upstream: TCAAGCCGAACTTTATAGACG	101
		downstream: ATGTGGTCAACTTCAATGTGG	
*VEGF*	JF831364.1	upstream: CTGTTTCTCTTGAGGGCAATC	126
		downstream: TATGGGAGGGTAGGGTGAG	
*FGF-2*	XM_005666885.1	upstream: TGCTATGAAGGAAGATGGAAG	105
		downstream: CTCGACCGGTAAGTATTGTAG	
*THBS1*	NM_011580.3	upstream: TCGACTGTGAGAAGATGGAGAA	112
		downstream: GTTGTCAAGGGTGACAAAGACA	
*ADAMTS*	JQ065373.1	upstream: CTCCTACCTTTCTTTCCTC	123
		downstream: TCTTGTTCTGGCATTACATC	
*TIMP*	NM_001145985.1	upstream: CGGAGGAAAGAAGGAGTA	190
		downstream: GGAGATGTAGCAGGGGAT	
*IGF-1*	NM_214256.1	upstream: AAGAAGGGTCACAACAAG	176
		downstream: CAAACCAAGAGAAGAAAG	
*IGF-2*	X56094.1	upstream: TGGCATCGTGGAAGAGTG	166
		downstream: CCAGGTGTCATAGCGGAA	
*IGFBP2*	HQ432890.1	upstream: ATGTCAGGCTAGTCTCTC	124
		downstream: TGGTATGTAACTTGGGGA	
*IGFBP5*	NM_214099.1	upstream: TCCAGTACGAAATCAAGC	114
		downstream: TTCCTCCGATGTCCAGCG	
*FN1*	XM_003133641.2	upstream: AATTGGCTTGGTCTGTAT	104
		downstream: CGGTCGTGATGGTATGTG	
*Cyclin D1*	XM_003468321.2	upstream: GAAATCAAGCAGATAAAG	178
		downstream: CGATGAAGTCACAGAGCG	
*P27* ^*kip*^	NM_214316.1	upstream: TGCCTTTAATTGGGTCTC	158
		downstream: GTTGGCTCTTTTGTTTTG	
*BCL 2*	NM_214285.1	upstream: CATGCGTATTTATATTTG	112
		downstream: CTCTGCTGCTTGCTGCTA	
*caspase-3*	NM_214131.1	upstream: ATGTCAGGCTAGTCTCTC	124
		downstream: TGGTATGTAACTTGGGGA	
*FOXL2*	NM_001244665.1	upstream: TGGCATCGTGGAAGAGTG	166
		downstream: CCAGGTGTCATAGCGGAA	
*c-FOS*	NM_001123113.1	upstream: TGGCATCGTGGAAGAGTG	166
		downstream: CCAGGTGTCATAGCGGAA	
*CEBP*	AY207000.1	upstream: AGACGCAGCATAAGGTCC	104
		downstream: CTTGAACAAGTTCCGCAG	
*INH*α	DQ356013.1	upstream: AGACCTTCGTGGTTATTT	108
		downstream: TGGCTACAGCTCTGATTC	
*INH*β	NM_001164842.1	upstream: TAATGGAATAACAAATGG	164
		downstream: AGGCAGAAGGAAGGGAGA	

### Statistical analysis

Differences between immunized and control groups, in terms of anti-inhibin α-subunit titer and concentrations of E2, were analyzed by one-way analysis of variance (ANOVA). Differences in gene and protein expression in granulosa cells were analyzed by ANOVA. The means were compared using the least significant difference method. All values we expressed as mean +/− SEM. All statistical analyses were performed with SAS software Version 8.01 (SAS Institute Inc.; Cary, NC, USA).

## Results

### E2 secretion

**Figure 1:**
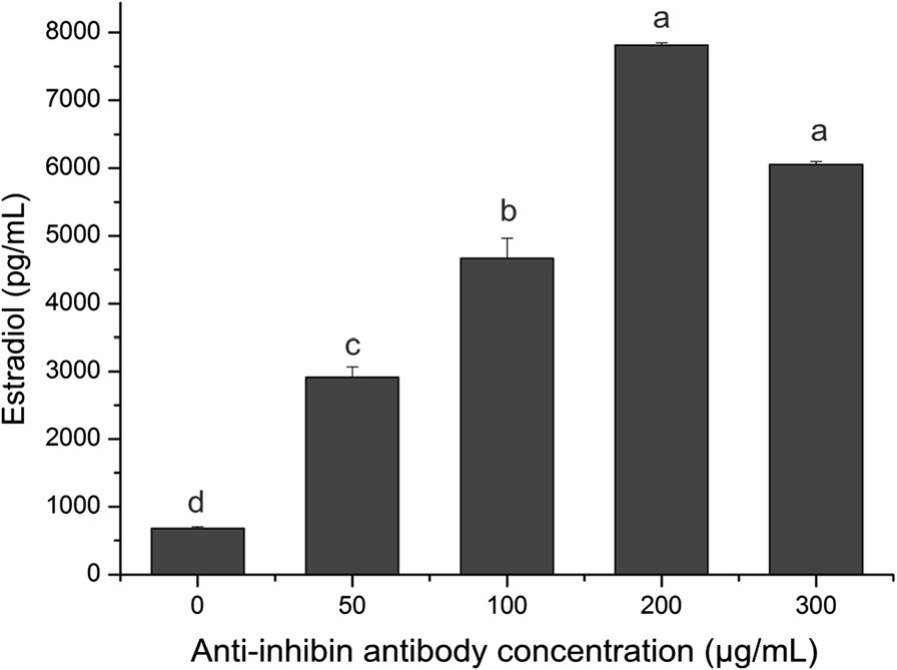
**Estradiol concentration by cultured porcine granulosa cells in response to anti-inhibin** **α-subunit antibody treatment at varying concentrations (0, 50, 100, 200, 300** **μg/mL).** Each value (plus SEM) represents the average of data from 6 independent culture experiments, each with 3 replicate wells. Means marked with different letters are significantly different (a-b, b-c, c-d, c-e, d-e: *P* < 0.01).

**Figure 2:**
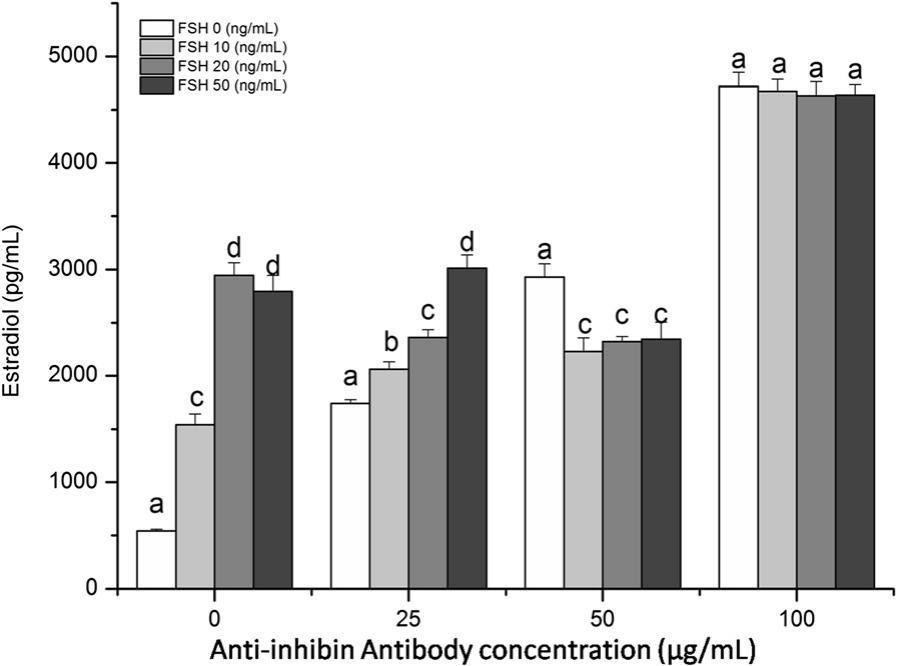
**Estradiol concentration by cultured porcine granulosa cells in response to treatments of varying concentrations of anti-inhibin** **α-subunit antibody (0, 25, 50, and 100** **μg/mL) and FSH (0, 10, 20, and 50 ng/mL).** Each value (plus SEM) represents the average of data of 6 independent culture experiments, each with 3 replicate wells. Means marked with different letters are significantly different (a-b, b-c: *P* < 0.05; a-c, a-d, b-d, c-d: *P* < 0.01).

Treatment with anti-inhibin α-subunit antibodies increased E2 concentrations in conditioned supernatants from the granulosa cell culture in a dose-dependent manner. The highest E2 concentration was 7814.68 pg/mL following treatment with 200 μg/mL antibody. E2 concentration decreased further in the presence of a higher (300 μg/mL) antibody concentration (Figure 1). When both anti-inhibin α-subunit antibody and FSH were added to the culture medium, we observed an interaction between the two treatments. Without the antibody, FSH treatment led to a dose-dependent increase in E2 secretion, which plateaued at 3000 pg/mL at FSH concentrations of 20 ng/mL. The anti-inhibin α-subunit antibody appeared to attenuate the stimulatory effect induced by FSH, especially at the concentrations of 25 and 50 g/mL. But at 100 g/mL antibody concentration, inclusion of varying levels of FSH into the medium did not affect E2 concentrations, which were indifferent from that by anti-inhibin α-subunit antibody treatment alone (Figure 2).

### Cell proliferation

**Figure 3:**
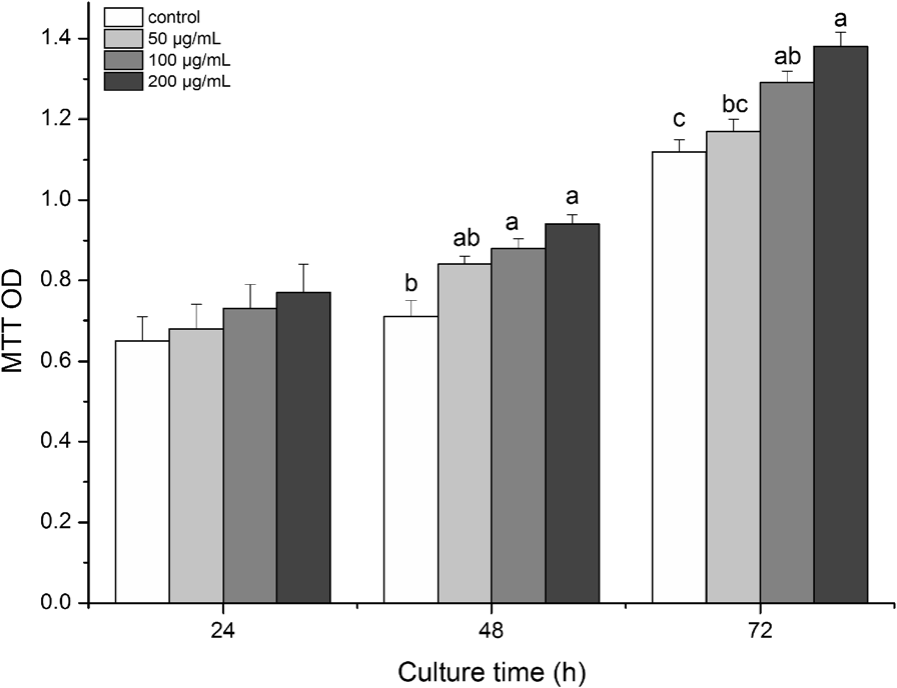
**Proliferation (represented by the MTT OD value) of porcine granulosa cells following culture for varying times and in response to different concentrations (0, 50, 100, and 200** **μg/mL) of anti-inhibin** **α-subunit antibody treatment.** Each value (plus SEM) represents data of 6 independent culture experiments, each with 3 duplicate wells. Means marked with different letters are significantly different (a-b, b-c: *P* < 0.05; a-c: *P* < 0.01).

As an indirect measure of cellular proliferation, the results from the MTT assay revealed that treatment with an anti-inhibin α-subunit antibody stimulated granulosa cell proliferation in a dose- and time-dependent manner. After 24 h of culture, the antibody slightly, but not significantly, increased the formazan OD value. At 48 and 72 h, inclusion of the anti-inhibin α-subunit antibody significantly (*P* < 0.05) increased the formazan OD value, indicating an increased rate of cellular proliferation (Figure 3).

### Protein phosphorylation

**Figure 4:**
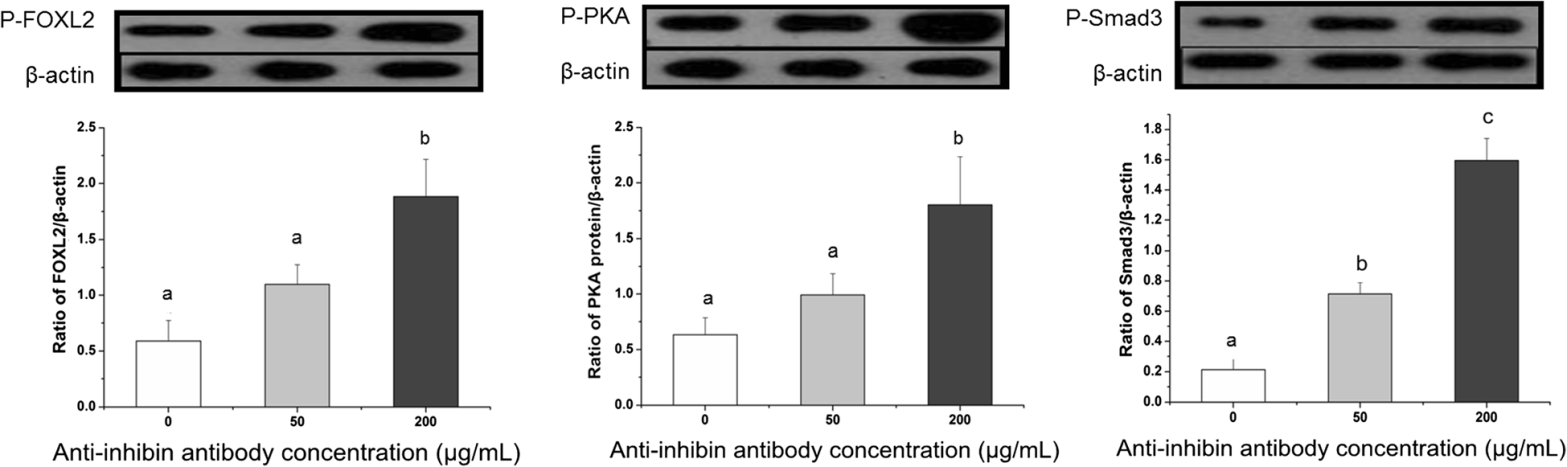
**Expression levels of phosphorylated FOXL2, PKA, and Smad3 proteins in cultured porcine granulosa cells in response to varying concentrations (0, 50, and 200** **μg/mL) of anti-inhibin** **α-subunit antibody treatment, normalized to** **β-actin expression.** Each value (plus SEM) represents the average data of 4 independent culture experiments, each with 2 duplicate wells. Means marked with different letters are significantly different (a-b: *P* < 0.01).

As shown in Figure 4, treatment of granulosa cells with an anti-inhibin α-subunit antibody increased the levels of phosphorylated Smad3, PKA, and FOXL2 relative to the level of the housekeeping protein β-actin.

### Gene expression

**Figure 5:**
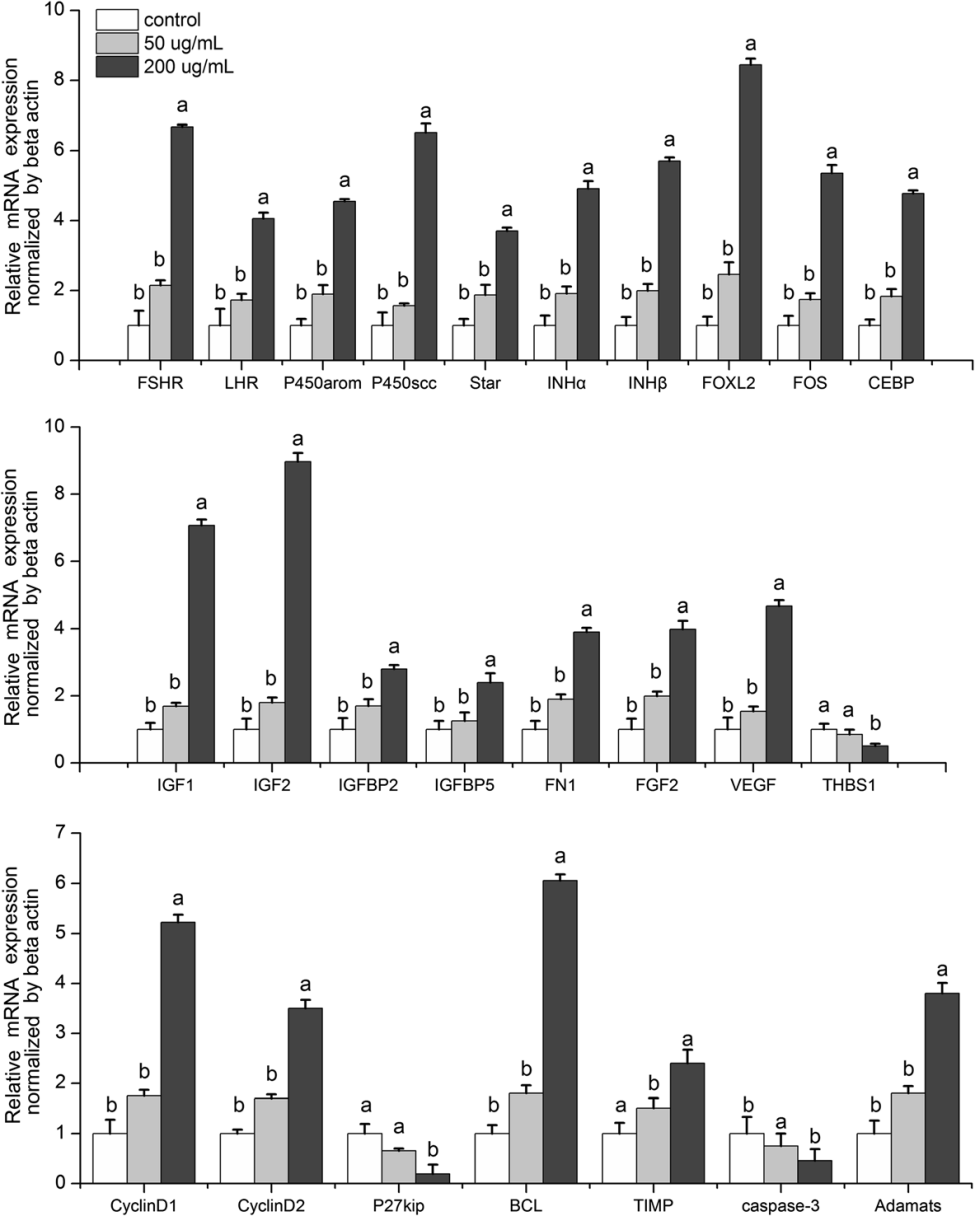
**mRNA expression levels of cultured porcine granulosa cells in response to varying concentrations (0, 50, and 200** **μg/mL) of anti-inhibin** **α-subunit antibody treatment, normalized to** **β-actin expression.** Each value (plus SEM) represents the average of data from 6 independent culture experiments, each with 3 duplicate wells. Means marked with different letters are significantly different (a-b: *P* < 0.05; a-c, a-d, b-c, c-d: *P* < 0.01).

Expression levels of the 26 genes assayed exhibited either up- or downregulation following anti-inhibin α-subunit antibody treatment during granulosa cell culture (Figure 5). Expression of genes involved in the regulation of sex hormone secretion (*FSHR, LHR, StAR, P450arom,* and *P450scc*) was upregulated following anti-inhibin α-subunit antibody treatment. The same was true for growth factors and related factors (*IGF1*, *IGF2*, *VEGF*, *FGF2*, *IGFBP2*, *IGFBP5*, *IHN*α, and *IHN*β), cell proliferation- and cell cycle-regulating genes (*FN1*, cyclinD1, and cyclinD2), anti-apoptotic or anti-atresia genes (*BCL2*, *TIMP*, and *ADAMTS*), and genes involved in follicular development, as well as function-related transcription factors (*FOXL2, FOS, CEBP*). Downregulated genes included *THBS1*, the cell cycle regulatory factor *p27*^*kip*^, and the gene encoding the apoptotic protein caspase-3.

## Discussion

This study investigated the impact of anti-inhibin α-subunit antibody treatment on granulosa cell function to determine the mechanisms of enhanced ovarian follicular development observed following inhibin immunization. The results of this study showed that treating cultured porcine granulosa cells with an anti-inhibin α-subunit antibody significantly augmented cell function in terms of E2 secretion and cell proliferation. These responses were due to significant changes in the expression patterns of genes coding for proteins associated with steroid hormone synthesis, transcription factors, and cell apoptosis, growth, and proliferation. These results suggest that treatment with an anti-inhibin α-subunit antibody has widespread effects on gene expression in granulosa cells.

Inhibin is a negative regulator of ovarian follicular development, due to its inhibitory effect on pituitary gonadotropin FSH secretion [13]. Furthermore, inhibin antagonizes the stimulatory effect of activin on follicular development [5,14]. Inhibin precursors have been found to compete with FSH binding at its receptor, FSHR, which reduces FSHR signaling and subsequent FSH stimulation of granulosa cell function [15]. In animal models, immunization against inhibin has been shown to stimulate the secretion of pituitary FSH, ovarian activin, and E2, leading to enhanced follicular development and supra-normal ovulation [6,10,16-18]. Enhanced ovarian follicular function is illustrated by follicular growth and increased hormone secretion capacity. Both these activities are affected by pituitary FSH, growth factors secreted by granulosa cells, and transforming growth factor family members, including inhibin [4,19-21]. The effects of these factors are mediated by complex intracellular signal pathways that induce protein phosphorylation, leading to enhanced transcription and gene expression of key molecules in granulosa cells.

In numerous animal studies, immunization against inhibin or its α-subunit stimulated ovarian follicle development and enhanced E2 secretion, which was frequently accompanied by enhanced pituitary FSH secretion [10,22,23]. By utilizing an *in vitro* cell culture method, which excluded interference from pituitary-secreted FSH, we were able to elucidate the regulatory effects exerted solely by inhibin on granulosa cells. For example, immunoneutralization of inhibin by the addition of an anti-inhibin α-subunit antibody into the cell culture medium led to a significant increase in E2 secretion in the present study. This result is consistent with the enhanced E2 secretion observed following the treatment of bovine granulosa cells with an anti-inhibin α-subunit antibody, as well as its attenuation following treatment with inhibin A [12]. Furthermore, the present study showed that anti-inhibin α-subunit treatment led to a significant increase in E2 concentration in the culture medium, reaching 7814.68 pg/mL, which is similar to the level observed in the previous bovine study [12]. Although neither activin nor inhibin concentration was measured in the cell culture medium, it is expected that both would be synthesized and secreted by granulosa cells, as mRNA for both the inhibin-α and -β subunits was detected and upregulated by anti-inhibin α-subunit treatment. Thus, the effects of inhibin immunoneutralization should be realized by a reduction in the inhibin/activin ratio in the culture system, which should reduce the antagonizing effect induced by inhibin, and thereby enhance activin binding to the ACTRII receptor. The enhanced activin stimulation will promote E2 secretion by cultured granulosa cells. This hypothesis is supported by the finding that anti-inhibin α-subunit treatment increased Smad3 phosphorylation, an intracellular ACTR signal transduction molecule, in cultured porcine granulosa cells in the present study.

Furthermore, an interaction between the anti-inhibin α-subunit antibody and FSH was observed in the regulation of E2 secretion by granulosa cells. FSH stimulation of E2 secretion in cultured porcine granulosa cells was augmented by anti-inhibin α-subunit antibody treatment at a concentration of 25 μg/mL. When the concentration was increased (50 μg/mL), the effect of FSH was compromised, resulting in a reduction in E2 concentration to almost 80% of the maximal level. Although this result appears highly unlikely, it is nevertheless supported by previous findings showing that high (100 ng/mL) and low (3–30 ng/mL) concentrations of activin respectively prevented and enhanced FSH receptor [24] and aromatase activity [25] in cultured rat granulosa cells. Our results are also in line with the impaired development of cultured rat follicles when cells were co-stimulated with FSH and activin. The impaired FSH effect was considered to have been caused by inhibin production in the rat study [17]. Thus, under very high (100 μg/mL) concentration of anti-inhibin antibody, which readily neutralizes *de novo* secreted inhibin, E2 secretion was not impaired by the addition of different FSH concentrations. In the present study, we did not measure inhibin concentration in the culture medium, because the antibody could interfere with the results, making it difficult to determine the effect on E2 secretion by *de novo* secreted inhibin. Nevertheless, it also appears that the effect of FSH on E2 secretion is masked when a high concentration of anti-inhibin antibody is present. Animal experiments have consistently shown that enhanced ovarian follicular E2 secretion is regulated by both anti-inhibin α-subunit and FSH [10,22,23]; this same effect could be equivalent to that brought about by the intermediate, but not high, levels of antibody and FSH used in this study. Enhanced E2 secretion could also arise because of a higher number of granulosa cells. Previous findings showed no effect of inhibin treatment on the proliferation of cultured granulosa cells [26,27], which makes the role of inhibin in granulosa cell proliferation inconclusive [5]. Nevertheless, the use of an anti-inhibin α-subunit antibody in the present study led to enhanced cell proliferation. This clearly suggests that inhibin may exert an inhibitory effect on granulosa cell proliferation. Such an effect could be mediated by antagonizing the effects of activin, which (alone or in combination with FSH) can exert profound promoting effects on the proliferation of cultured bovine and rat granulosa cells [21,27]. Thus, the use of an anti-inhibin α-subunit antibody in this study allowed us to overcome the disadvantage of using inhibin directly.

In this study, immunoneutralization of inhibin led to the activation of several transcription factors such as phosphorylated Smad3, PKA, and FOXL2 proteins, and mRNA expression of *CEBP* and *c-FOS*. Immunoneutralization of inhibin can increase the activin/inhibin ratio or create a state of activin ‘tone’ [5,21] in the cell culture medium. As a result, through ACTR signaling from *de novo* synthesized and secreted activin, the expression of phosphorylated Smad3 is elevated in cultured granulosa cells. Enhanced Smad3-mediated signaling can upregulate gene expression of *FSHR* and its signal pathway molecule PKA [28], and also potentiates the ovarian response to FSH stimulation [29]. Moreover, phosphorylation of PKA was enhanced in this study, although no FSH was added to the cell culture medium. Thus, enhancement of both the ACTR and FSHR signaling pathways should strengthen granulosa cell proliferation and function. Furthermore, phosphorylation of FOXL2, the earliest marker of ovarian differentiation and a key regulator of ovarian development and function [30-32], was upregulated by treatment with anti-inhibin α-subunit antibody, or increased activin ‘tone’. Thus, PKA through interaction with CEBP, together with Smad3, FOXL2, and c-FOS, modulate the expression of key genes involved in granulosa cell differentiation as well as steroidogenesis, such as *StAR*, *P450scc*, *P450Cyp17*, and *Cyp19A1* [33-38]. For example, in the promoter region of the aromatase gene, *Cyp19A1*, there exist *CRE-like*, *FOXL2*, and *Smad3* response elements in the proximal promoter, and *c-fos* AP family factor and *CRE* response elements in the distal promoter [34,35,39,40]. Furthermore, FOXL2 interacts with Smad3 and another transcription factor, CATA4, to regulate granulosa cell viability and apoptosis; in particular, FOXL2 inhibits cell cycle progression [41,42] and induces apoptosis, while the latter two factors suppress apoptosis [42]. PKA, via its downstream target CREB, stimulates granulosa cell proliferation [35]. It is clear that synergistic and complex regulation mediated by these transcription factors contribute to enhanced E2 secretion and cell proliferation following immunoneutralization of inhibin.

Under the regulation of these transcription factors, changes in granulosa cell functions are fulfilled by up- or downregulating the expression of relevant genes. Therefore, enhanced E2 synthesis induced by anti-inhibin α-subunit treatment was accompanied by upregulated expression of steroidogenic genes *StAR, P450scc,* and P450arom, as well as *FSHR, LHR, IHN*α*,* and *IHN*β. Since the balance of cyclinD2 and *p27*^*kip*^ expression determines cell cycle progression, meiosis, and proliferation [43], the upregulation of the cell cycle progression factors cyclinD1 and cyclinD2, and the downregulation of the anti-proliferation gene *p27*^*kip*^ should favor cell division or proliferation, as represented by the higher MTT values of cells treated with an anti-inhibin α-subunit. Likewise, antibody treatment also upregulated the expression of *IGF-I*, *IGF-II*, and the genes encoding their binding proteins, *IGFBP2* and *IGFBP5*. The expression of the former two genes also promotes granulosa cell proliferation and E2 secretion, while the latter two binding proteins play counter roles and could induce apoptosis of cells and follicle atresia [4,19]. Upregulation of anti-apoptotic genes (*BCL2*, *TIMP*, and *ADAMTS*) [44-46], and downregulation of pro-apoptotic genes (caspase-3) will further augment granulosa cell viability and cell density following treatment with anti-inhibin α-subunit antibody. This will function to further uphold E2 secretion. Some genes involved in vascularization or angiogenesis, namely, *FGF2* and *VEGF*, were also upregulated, while *THBS1* expression was downregulated by treatment with anti-inhibin α-subunit antibody. THBS1 inhibits VEGF expression in the ovary via the low-density lipoprotein receptor-related protein-1 [47]. Moreover, fibronectin 1, which plays a vital role in extracellular matrix formation [48], was regulated in anti-inhibin α-subunit antibody-treated cells. These results indicate that anti-inhibin α-subunit antibody-treated granulosa cells also synthesize and secrete factors and substances that can promote follicular growth, angiogenesis, and extracellular matrix remodeling, while simultaneously resisting apoptosis or follicle atresia.

## Conclusions

These results suggest that immunoneutralization of inhibin bioactivity, through the augmentation of activin and gonadotrophin receptor signaling pathways and regulation of gene expression, permits the development of healthy and viable granulosa cells. These molecular mechanisms help to explain the overstimulation of ovarian follicular development observed following inhibin immunization in animal models.

## Abbreviations

Cyp19A1: Cytochrome P450, family 19, subfamily A, polypeptide 1
P450arom: P450 aromatase
P450scc: Cholesterol side-chain cleavage cytochrome P450
FSH: Follicle-stimulating hormone
FSHR: Follicle-stimulating hormone receptor
LHR: Luteinizing hormone receptor
StAR: Steroidogenic acute regulatory protein
VEGF: Vascular endothelial cell growth factor
FGF2: Fibroblast growth factor 2
THBS1: Thrombospondin 1
TIMP: Tissue inhibitor of metalloproteases
ADAMTS: A disintegrin and metalloproteinase with thrombospondin motifs
IGFs: Insulin-like growth factors
IGFBPs: Insulin-like growth factor-binding proteins
BCL: B-cell CLL/lymphoma 2
p27^kip^: Cyclin-dependent kinase inhibitor 1B
FOS: FBJ murine osteosarcoma viral oncogene homolog
FOXL2: Forkhead box L2
CEBP: CCAAT/enhancer binding protein
